# Preventing workplace mistreatment and improving workers’ mental health: a scoping review of the impact of psychosocial safety climate

**DOI:** 10.1186/s40359-024-01675-z

**Published:** 2024-04-08

**Authors:** Mustapha Amoadu, Edward Wilson Ansah, Jacob Owusu Sarfo

**Affiliations:** Department of Health, Physical Education and Recreation, University of Cape Coast, Cape Coast, Ghana

**Keywords:** Psychosocial safety climate, Workplace violence, Workplace abuse, Mental health

## Abstract

**Background:**

Work environment is rapidly evolving, unfortunately, it is also becoming increasingly hostile for workers due mostly to common psychosocial hazards. This situation is posing significant challenges for organisations to protect the psychological well-being of their workers. Hence, this review aims to map studies to understand the influence of psychosocial safety climate (PSC) on workplace mistreatment and mental health of workers.

**Methods:**

The guidelines outlined by Arksey and O’Malley were adopted for this review. PubMed, Scopus, Web of Science, JSTOR, Google and Google Scholar were searched for relevant papers. Only peer-reviewed studies that measured PSC using PSC-12, PSC-8 or PSC-4 were included in this review.

**Results:**

Thirty-eight studies met the inclusion criteria. This review found that PSC has a negative association with workplace mistreatment such as bullying, harassment, violence, discrimination and abuse. Further, PSC has a positive association with psychological well-being, personal resilience and hope. Low level organisational PSC also promotes psychological distress, stress, depression, cognitive weariness and emotional exhaustion. The buffering effect of PSC is well-established. Moreover, PSC mediates the association between health-centric leadership and workers’ psychological health problems. The inverse relationship between PSC and depressive symptoms was stronger for females than males.

**Conclusion:**

Organisations should prioritise training and development of supervisors to enhance their supportive skills, encourage respectful behaviour, encourage the use of resources promote open and bottom-up communication and provide guidance on conflict resolution. By promoting a high PSC context, organisations can create a culture that discourages mistreatment, leading to increased employee well-being, job satisfaction, and productivity.

**Supplementary Information:**

The online version contains supplementary material available at 10.1186/s40359-024-01675-z.

## Introduction

Work environment globally is rapidly evolving, but it is also becoming increasingly hostile for workers. Recent evidence from the International Labour Organisation (ILO) indicates that about 23% of workers have experienced violence and harassment at work, encompassing physical, psychological, and sexual abuse [1]. This disturbing statistic reveals that more than one in five people in employment have encountered workplace violence and harassment, posing significant challenges for organizations to protect the psychological well-being of their workforce. There is also a growing realization of the need to understand the influence of the psychosocial work environment on workplace mistreatment and mental health. The World Health Organisation (WHO) and ILO have jointly reported a global increase in occupational morbidity and mortality resulting from a poor psychosocial work environment [2, 3], emphasising the importance of exploring the concept of psychosocial safety climate (PSC).

PSC is an organisational culture that prioritises workers’ psychological health and safety at the workplace [4]. Thus, PSC refers to the shared perceptions of workers concerning workplace policies, practices, and procedures that are designed to protect and promote their psychological well-being [5]. It encompasses a range of organisational factors including leadership commitment to workers’ well-being, job design, organisational justice, social support and overall climate of trust and respect at the workplace [6]. A high PSC context emphasises the importance of fostering a psychologically healthy work environment, where workers feel safe, supported, valued, treated fairly and respected [7], thus, lowering the tendency of mistreatment of workers.

Workplace mistreatment refers to any form of harmful, abusive, or disrespectful behaviour that occurs in the work environment [1] This includes but not limited to bullying, harassment, violence, abuse and discrimination [1]. Workplace mistreatment has gained significant research and policy attention due to its detrimental effects on both workers and organizations [1]. Victims of workplace mistreatment often experience poor mental health, job dissatisfaction and impaired productivity [8]. Recognising the crucial role of the PSC in mitigating workplace mistreatment and protecting workers’ mental health has become a pressing concern for researchers, industries and policymakers [8]. For over a decade of research into PSC, identifying and synthesising studies that have explored PSC in reducing workplace mistreatment and improving mental health is noteworthy. Thus, this scoping review aims at mapping existing studies to provide a comprehensive understanding of the influence of PSC on workplace mistreatment and mental health on workers. The purpose is to make recommendations for future research and systematic reviews. This review will also help organisations, managers and policymakers to develop evidence-based strategies and interventions that promote a PSC work-context that fosters a respectful and supportive work environment and safeguard workers’ psychological well-being. Also, this review aims to provide evidence that is useful in promoting a healthy and decent workplace that eliminates all forms of workplace mistreatment and mental health stressors.

## Methods

The guidelines outlined by Arksey and O’Malley [9] were adopted for this scoping review: thus, identifying and stating the research questions, identifying relevant studies, studies selection, data collection, summary and synthesis of results and consultation. Therefore, we formulated research questions based on the Population, Concept and Context (PCC) framework. The following questions guided this scoping review:

1. What is the relationship between PSC and workplace mistreatment?
2. What is the relationship between PSC and mental health parameters?
3. What is the mediating and moderating role of PSC in improving workers’ mental health and reducing workplace mistreatment?

Search for relevant papers was conducted in four main databases (PubMed, Scopus, Web of Science, and JSTOR). Google and Google Scholar were explored for additional papers. Reference lists of eligible records were also checked for relevant papers. The authors created a search technique that used a combination of controlled vocabularies like Medical Subject Headings (MeSH). Keywords for each of the four major electronic databases (PubMed, Scopus, Web of Science and JSTOR) were also created to address the research questions and identify relevant literature. Table 1 presents the search strategy conducted in PubMed and other databases. The search strategies were informed by PCC. The context was not limited to a specific country or region since this review was given a global focus. The search strategy used in PubMed was then modified for search in other databases. The authors used three keywords in their search strategy (1) psychosocial safety climate, (2) workplace abuse and (3) mental health. The search for relevant papers started on March 12, 2023, and ended on July 1, 2023. Chartered librarians at the Sam Jonah Library, University of Cape Coast were consulted.

**Table 1:** Search Strategy Search strategy conducted in PubMed

#1	Search to identify psychosocial safety climate	Psychosocial safety climate*[MeSH Terms] OR psychological safety climate* OR and psychosocial safety culture* OR safety climate*
*#2*	Search to identify workplace abuse	Workplace mistreatment* [MeSH Terms] OR Workplace abuse OR psychosocial working conditions* OR workplace bullying* OR workplace mistreatment* OR workplace harassment* OR workplace harassment* OR Workplace bullying* OR Workplace victimisation* OR Workplace aggression* OR Workplace discrimination* OR Workplace sexual abuse* OR Workplace intimidation* OR Workplace violence* OR Workplace incivility* OR Workplace maltreatment*
*#*3	Search to identify mental health	Mental health* [MeSH Terms] OR psychological well-being* OR quality of work life* OR quality of life* OR emotional exhaustion* OR Depression* OR anxiety* OR mindfulness* OR Post-traumatic stress* OR mental distress* OR mental health* OR Emotional well-being* OR Mental well-being* OR Stress* OR Cognitive stress*
	Overall search strategy	#1* AND ***#*** **2 AND** ***#*** 3NOT animal* **(Filters activated: English, from 2010/01/01)**
**Search strategy in PubMed modified for search in Scopus**		
((TITLE-ABS-KEY (“psychosocial safety climate” OR “psychological safety climate” OR “psychosocial safety culture” OR “safety climate”)) AND (TITLE-ABS-KEY (“Workplace mistreatment” OR “Workplace abuse” OR “psychosocial working conditions” OR “workplace bullying” OR “workplace harassment” OR “Workplace victimisation” OR “workplace aggression” OR “workplace discrimination” OR “workplace sexual abuse” OR “workplace intimidation” OR “workplace violence” OR “workplace incivility” OR “workplace maltreatment”)) AND (TITLE-ABS-KEY (“Mental health” OR “psychological well-being” OR “quality of work life” OR “quality of life” OR “emotional exhaustion” OR “Depression” OR “anxiety” OR “mindfulness” OR “Post-traumatic stress” OR “mental distress” OR “mental health” OR “Emotional well-being” OR “Mental well-being” OR “Stress” OR “Cognitive stress”)) NOT TITLE-ABS-KEY (“animal”)) AND (LIMIT-TO (LANGUAGE, “English”)) AND (LIMIT-TO (PUBYEAR, 2010–2023))		
**Search strategy in PubMed modified for search in Web of Science**		
(TS=(“psychosocial safety climate*” OR “psychological safety climate*” OR “psychosocial safety culture*” OR “safety climate*”)) AND (TS=(“Workplace mistreatment*” OR “Workplace abuse” OR “psychosocial working conditions*” OR “workplace bullying*” OR “workplace mistreatment*” OR “workplace harassment*” OR “workplace harassment*” OR “Workplace bullying*” OR “Workplace victimisation*” OR “Workplace aggression*” OR “Workplace discrimination*” OR “Workplace sexual abuse*” OR “Workplace intimidation*” OR “Workplace violence*” OR “Workplace incivility*” OR “Workplace maltreatment*”)) AND (TS=(“Mental health*” OR “psychological well-being*” OR “quality of work life*” OR “quality of life*” OR “emotional exhaustion*” OR “Depression*” OR “anxiety*” OR “mindfulness*” OR “Post-traumatic stress*” OR “mental distress*” OR “mental health*” OR “Emotional well-being*” OR “Mental well-being*” OR “Stress*” OR “Cognitive stress*”)) NOT TS=(“animal*”)) AND TS = (LANGUAGE, “English”)) AND TS= (PUBYEAR, 2010–2023))		
**Search strategy in PubMed modified for search in JSTOR**		
(ti:“psychosocial safety climate” OR ti:“psychological safety climate” OR ti:“psychosocial safety culture” OR ti:“safety climate”) AND (ti:“workplace mistreatment” OR ti:“workplace abuse” OR ti:“psychosocial working conditions” OR ti:“workplace bullying” OR ti:“workplace harassment” OR ti:“workplace discrimination” OR ti:“workplace violence” OR ti:“workplace incivility”) AND (ti:“mental health” OR ti:“psychological well-being” OR ti:“quality of work life” OR ti:“quality of life” OR ti:“emotional exhaustion” OR ti:“depression” OR ti:“anxiety” OR ti:“mindfulness” OR ti:“post-traumatic stress” OR ti:“mental distress” OR ti:“mental health” OR ti:“emotional well-being” OR ti:“mental well-being” OR ti:“stress” OR ti:“cognitive stress”) AND (ti: “Not animal”) AND (ti: Publication Year, “2010–2023”).		

NB: Searches in all databases were conducted from March 12, 2023 to July 1, 2023)

Mendeley software was used to remove duplicate records. Ten graduate students were trained and supervised by MA to screen titles and abstracts for full-text-eligible records. This was done to enhance efficiency in the screening process and allowed for a more thorough and expedited review of titles and abstracts to identify records eligible for full-text examination. Authors checked the reference list of full-text records to identify additional eligible records. Eligible full-text records were then screened independently by MA and JOS and supervised by EWA using the eligibility criteria presented in Table 2. Weekly meetings were used to resolve disagreements identified during the screening process.

**Table 2:** Eligibility criteria for screening search results and full-text records

**Inclusion criteria:**
1. The paper is written or published in the English language; 2. Only peer-reviewed articles; 3. The study should explore the psychosocial safety climate and workplace abuse and or mental health among the working population; 4. The study adopted or adapted the PSC-12, PSC-8, or PSC-4 to measure psychosocial safety climate or interview participants on PSC; 5. The study was conducted in any part of the world; 6. The study was published online in the year 2010 or later.
**Exclusion criteria**:
1. The paper was written or published in any other language other than English; 2. The paper is a conference paper, a letter to the editor, pre-print, grey literature, and commentaries; 3. The paper explored safety climate, physical safety climate, safety culture, or related constructs; 4. The paper did not adopt or adapt PSC-12, PSC-8 or PSC-4 in measuring psychosocial safety climate; 5. The paper was published before the year 2010 (Psychosocial safety climate was introduced in the year 2010); 6. Abstracts without full-text records; 7. The study was published online before the year 2010.

Data extraction was handled independently by two independent researchers (MA and JOS) and supervised by EWA. This was done to ensure that accurate and reliable data were extracted for this review. Disagreements during the data extraction phase were handled during weekly meetings. Authors extracted data on authors, the country where the study was conducted, year of publication, purpose of the study, study design, population, sample size, measure of PSC and study outcomes. Finally, thematic content analysis was conducted by the authors based on the research questions. The analysis involved identifying recurring themes relevant to the research questions. This process included organising and categorising data to extract meaningful patterns and insights from the extracted information. The search results, characteristics of reviewed studies and thematic analysis were presented.

## Results

### Search results

**Fig. 1:**
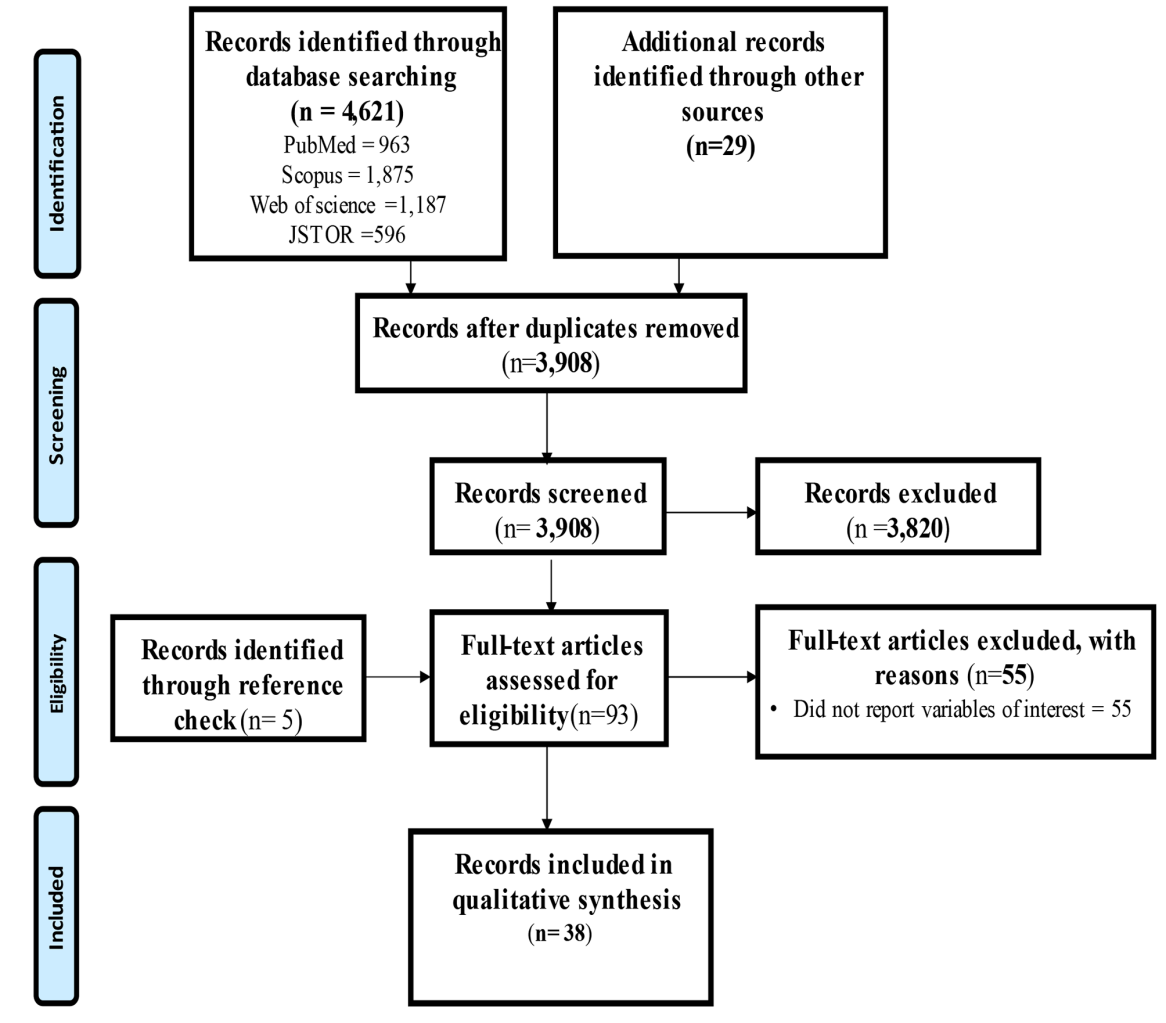
PRISMA flow diagram of search results and screening process

Search conducted in the four main databases produced 4,621 records and additional 29 records were retrieved from Google and Google Scholar. The Mendeley software was used to remove 742 duplicate records. After title and abstract screening, 3,820 records were removed because they were not relevant to this review. Additional 5 records were retrieved through reference checking of eligible studies and 93 full-text records were screened for eligibility. Finally, 38 full-text records were included in this scoping review and the remaining 55 full-text records were removed because they did not report on variables of interest. The search results and screening process is presented in Fig. 1.

### Characteristics of reviewed studies

**Fig. 2:**
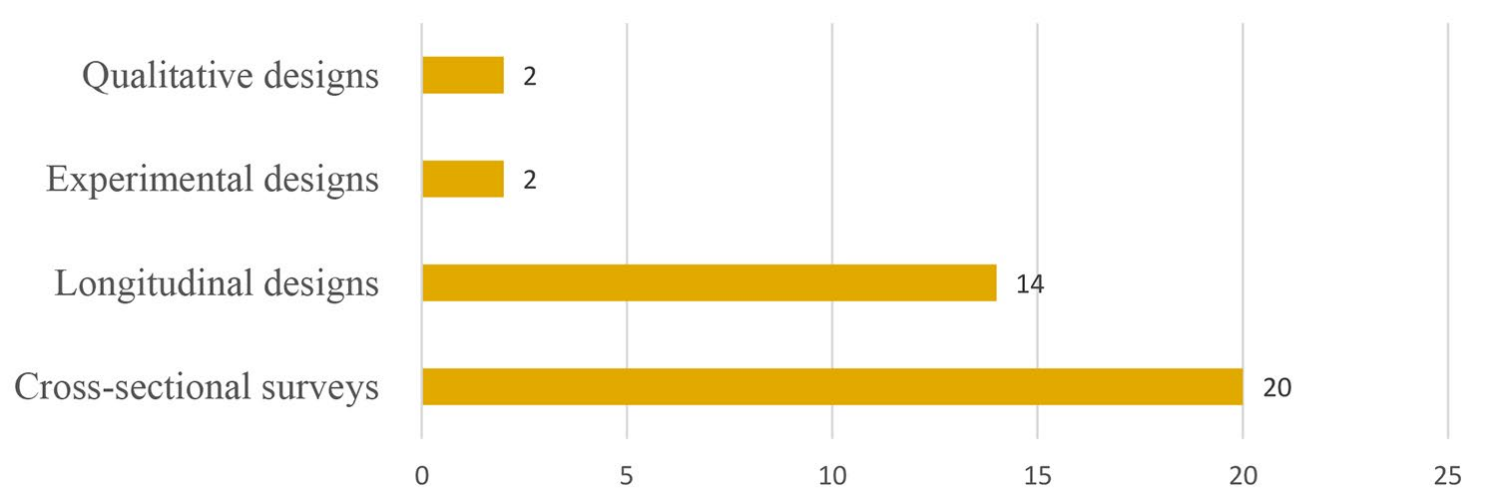
Study designs of reviewed studies

**Fig. 3:**
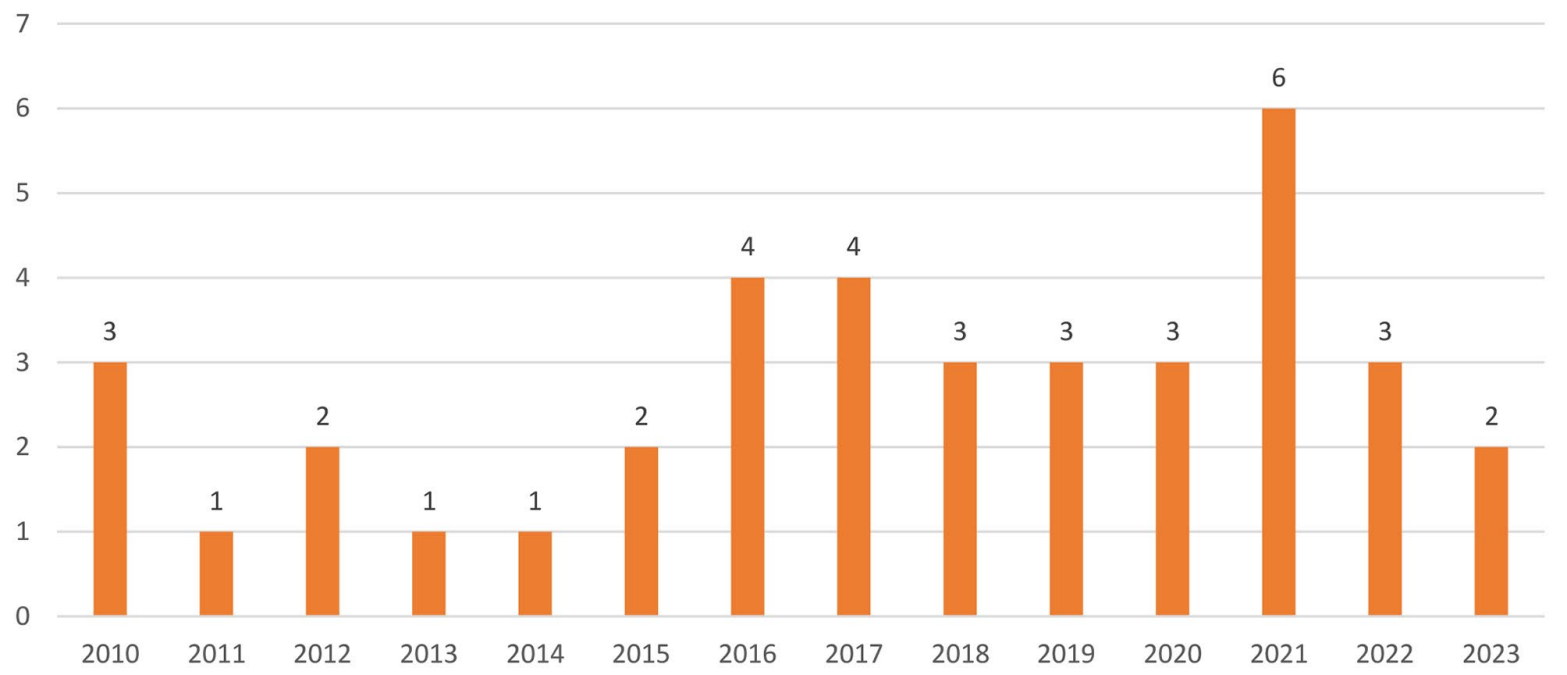
Year of publication of included studies

**Fig. 4:**
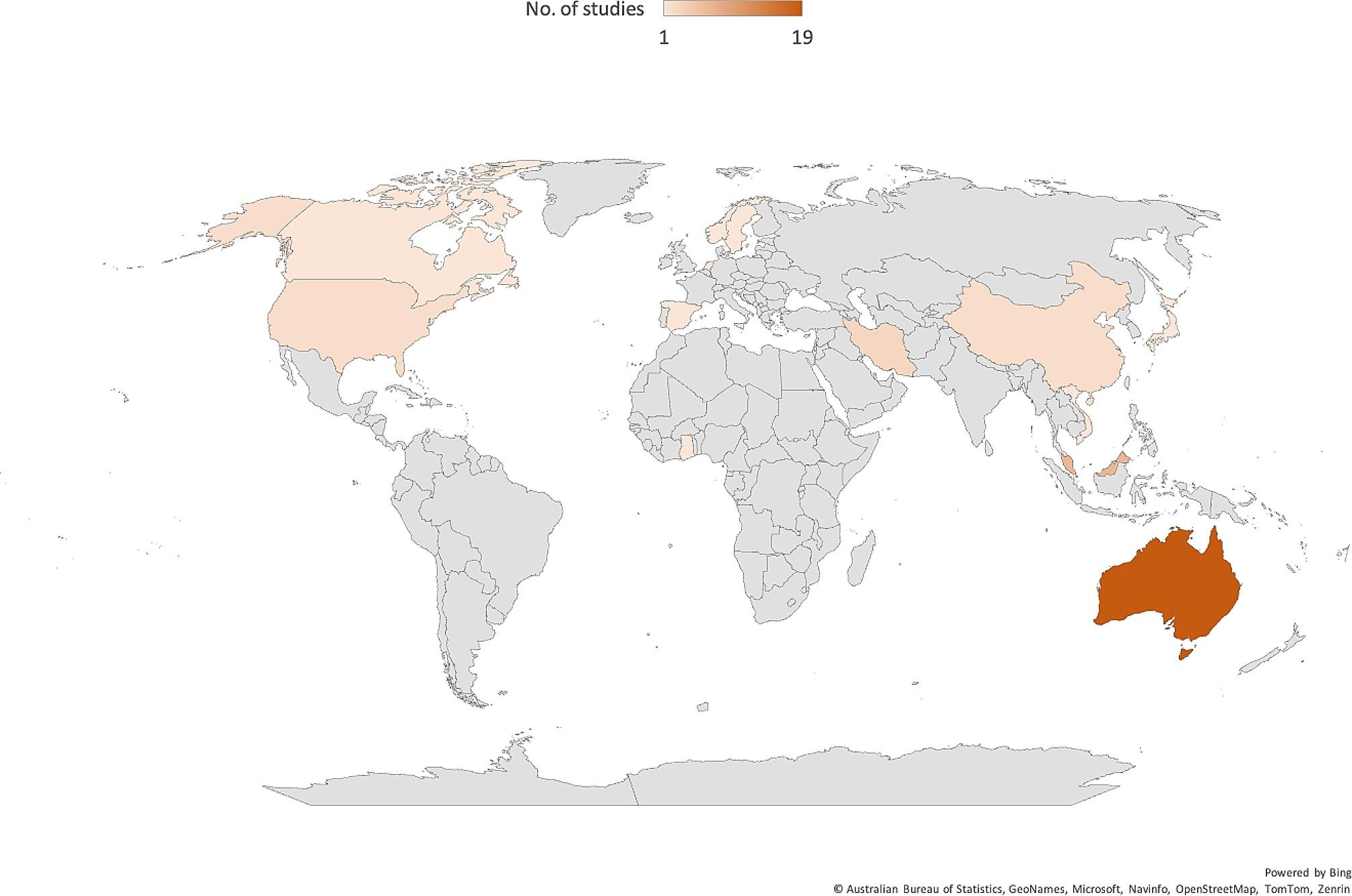
Map showing countries and continents where reviewed studies were conducted

**Fig. 5:**
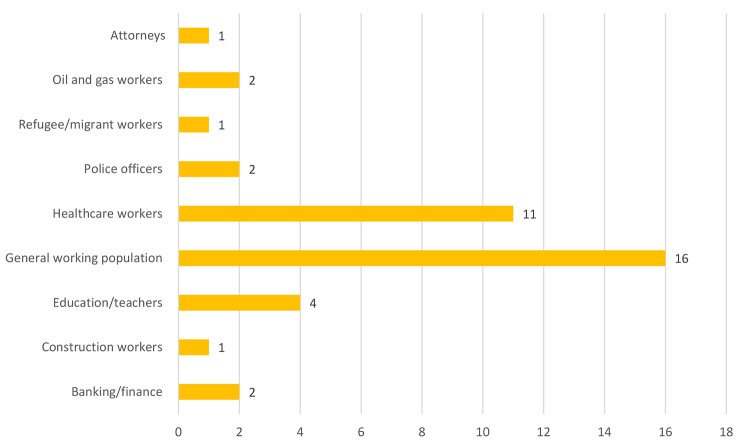
Occupational groups explored by reviewed studies

Reviewed studies collectively sampled 53,733 workers. A cross-sectional survey design was mostly used in conducting these studies (See Fig. 2 for details). A few (6) of the studies were published in 2021 (See Fig. 3 for details), with about half (19) conducted in Australia (See details in Fig. 4). Most of the studies we reviewed sampled general working population (16) and healthcare providers (11) (See details in Fig. 5). Characteristics of reviewed studies are presented in Supplementary File (Table S1).

### Influence of PSC on workplace mistreatment

Evidence indicates that PSC has a direct and significant influence on workplace mistreatment. For instance, reviewed studies reported that a high PSC work context provides a favourable work environment that helps eliminates or reduces workplace bullying among workers [10–17]. Also, workplace violence [8, 10] and abuse [18] are common in a low PSC context. In addition, studies further highlighted that workplace harassment [10, 11] and discrimination [18] are less common or eliminated in a high PSC context.

### Influence of PSC on workers’ mental health

Evidence established that PSC directly improves workers’ mental health. For example, evidence is consistent that high PSC context improves psychological well-being [14, 19–21] and reduces psychological distress [4, 18, 22–29]. Furthermore, it is indicated that low PSC work exposes workers to emotional exhaustion [4, 16, 22, 25, 30–32], stress [33, 34], cognitive weariness [35] and depression [23, 36–38]. For instance, a study reported that the inverse relationship between PSC and depressive symptoms was stronger for females than males [36]. Moreover, a high PSC context makes workers more assertive [39] and resilient [40] and presents opportunities for hope [40]. We present thematic analysis of the influence of PSC on workplace mistreatment and mental health in Table 3.

**Table 3:** Themes generated from reviewed studies

Main theme	Specific factors	Definitions	Authors
Workplace mistreatment	Workplace harassment	It refers to any unwanted or unwelcome behaviour, whether verbal, physical, or visual, that creates a hostile, intimidating, or offensive work environment, typically targeting a worker or a group of workers based on their protected characteristics such as gender, race, religion, or disability.	[10, 11]
	Workplace violence	Refers to any act or threat of physical violence, aggression, or intimidation that occurs within or related to the work environment, posing a risk to the safety and well-being of workers	[8, 10]
	Workplace bullying	Involves persistent, unwanted, and aggressive behaviour directed towards an individual or a group, typically involving the misuse of power or authority, with the intention to intimidate, humiliate, or undermine the target, creating a hostile and harmful work environment.	[10–17]
	Workplace abuse	Involves mistreatment of employees through acts of physical, psychological, or emotional harm, often involving the misuse of power or authority, resulting in the degradation, humiliation, or exploitation of the individuals targeted.	[18]
	Discrimination	It involves the denial of equal opportunities, unequal pay, biased decision-making, or creating a hostile work environment based on these protected attributes.	[18]
Mental health	Psychological well-being	It refers to a worker’s overall state of mental health and happiness, characterised by positive emotions, a sense of purpose and fulfillment, good self-esteem, and a strong ability to cope with life’s challenges.	[14, 19–21]
	Emotional exhaustion	It is a state of extreme fatigue and depletion of emotional resources, often resulting from prolonged periods of stress, excessive workload, and emotional demands. It is characterised by feelings of emotional drain, reduced motivation, and a sense of being emotionally overwhelmed or drained.	[4, 16, 22, 25, 30–32]
	Psychological distress	It involves experiencing negative emotions, such as sadness, fear, or worry, along with symptoms like sleep disturbances, difficulty concentrating, irritability, or a sense of hopelessness.	[4, 18, 22–29]
	Stress	It refers to the body and mind’s response to demanding or challenging situations that can have negative effects on physical and mental well-being, leading to symptoms such as irritability, fatigue, difficulty concentrating, and various health problems.	[33, 34]
	Depression	It is a mental health disorder marked by persistent sadness, loss of interest, and impaired daily functioning.	[23, 36–38]
	Cognitive weariness	Cognitive weariness refers to a state of mental exhaustion or fatigue that arises from prolonged cognitive effort, such as intense mental work, decision-making, or problem-solving. It involves a feeling of mental drain, reduced cognitive functioning, and a decreased ability to concentrate or think clearly.	[35]
	Assertive	Being assertive refers to expressing one’s thoughts, feelings, and needs in a confident and direct manner, while respecting the rights and boundaries of others	[39]
	Personal resilience	It involves the capacity to maintain mental strength, emotional well-being, and a positive outlook in the face of difficulties. Resilient workers can effectively cope with setbacks, learn from experiences, and develop strategies to navigate through adversity, ultimately promoting their overall well-being and ability to thrive.	[40]
	Hope	Hope is an optimistic and positive state of mind characterised by the belief that things can improve or desired outcomes can be achieved. It involves having confidence, aspirations, and a sense of possibility for the future, even in challenging circumstances.	[40]

### Mediation and the buffering effect of PSC

The literature consistently confirms the buffering effect of PSC on various outcomes related to worker well-being and mistreatment. Yulita et al. [7]for example, found that in a high PSC context, job resources had a stronger impact on reducing psychological distress. Similarly, Lawrie et al. [41] demonstrated positive impact of job control on worker mindfulness which is enhanced in a high PSC work environment. Besides, Siami et al. [40] revealed that the association between supportive leadership and personal hope is strengthened in the presence of a high PSC. Hall et al. [42] showed that the effect of job demands on depression is diminished when workers perceive high PSC. Additionally, Loh et al. [43] found a negative impact of emotional demands on psychological well-being that is mitigated in a high PSC context.

Furthermore, it is indicated that the adverse association between workplace bullying, harassment, and psychological well-being is attenuated when workers perceive high PSC [11]. Thus, PSC has the capacity to reduce the impact of workplace bullying on post-traumatic disorder and work engagement [12, 44] Kwan et al. [39] revealed that the positive association between bullying and neglect is diminished when a high PSC is perceived by workers. Moreover, PSC moderates the associations between role conflict and workplace bullying [17], role ambiguity and workplace bullying [17] and stigma and workplace bullying [15]. While a limited research attention is given, a study reported that PSC mediates the association between health-centric leadership and workers’ psychological health problems [26]. These findings collectively emphasize the crucial role of PSC in mitigating the negative consequences of mistreatment and enhancing workers’ well-being.

### Workplace-specific findings

The findings from various studies reveal workplace-specific outcomes related to Psychosocial Safety Climate (PSC). In the education sector in Australia, PSC was associated with a reduction in psychological distress and emotional exhaustion among education workers [4]. Similarly, in Malaysia, among police officers, PSC buffered the effect of job resources on psychological distress [7]. Healthcare workers in China experienced a decrease in workplace violence in the presence of a positive PSC [8]. For the general working population in Australia, PSC was linked to a decrease in harassment, violence, and bullying [10]. In diverse settings, including police officers, the general working population, and refugees in Australia, PSC demonstrated a consistent negative association with workplace mistreatment such as bullying, harassment, violence, discrimination, and abuse [12, 13, 18]. Healthcare workers in both Australia and Malaysia reported improved psychological well-being in the presence of a high PSC context [19]. Construction workers in China showed enhanced mental well-being [20]. Various workplace settings, such as attorneys in the USA and oil and gas workers in Malaysia, exhibited a decrease in psychological distress with a positive PSC [27, 28]. Notably, healthcare workers across different countries, including Iran, Australia, and Canada, experienced positive outcomes such as decreased emotional exhaustion and stress, emphasizing the universal impact of PSC in healthcare settings [22, 30, 35].

## Discussion

This review found that PSC has a negative association with workplace mistreatment such as bullying, harassment, violence, discrimination and abuse. Furthermore, we found that PSC has a positive association with psychological well-being, personal resilience and hope. Moreover, PSC has a negative association with psychological distress, stress, depression, cognitive weariness and emotional exhaustion. The buffering effect of PSC is well-established in the literature.

### Influence of PSC on workplace mistreatment

PSC has a negative association with workplace mistreatment. A high PSC work environment indicates that managers and supervisors are perceived as supportive, approachable and caring towards their workers. In such a work context, workers are more likely to feel protected, valued and respected [5]. Furthermore, such work context acts as a deterrent to workplace mistreatment including bullying and harassment because workers are more assertive at work [18]. Furthermore, when workers perceive their supervisors as supportive, they are likely to develop trust and respect among workers and towards their supervisors. This trust and respect may lead to positive interpersonal relationships between supervisors and workers, fostering a sense of fairness, partnership, and open communication [10, 11]. In such a work context, discrimination and mistreatment are less likely to occur, since they contradict the principles of trust and respect. In a high PSC context, managers and supervisors are expected to exhibit positive behaviours to serve as role models for their colleagues and subordinates. When managers and supervisors exhibit respectful and inclusive behaviours, it serves as precedents for acceptable conduct at the workplace, reducing the occurrence of mistreatment [8, 10]. In a high PSC work context, bottom-up communication is encouraged and supervisors are more likely to intervene and address workplace mistreatment, provide training on respectful behaviours and establish mechanisms for reporting incidents [5]. These communication and conflict resolution mechanisms do not only deter mistreatment but also provide a sense of security for workers.

### Influence of PSC on mental health

PSC has a positive association with psychological well-being. In a high PSC context, workers are trained and encouraged to utilise essential resources capable of helping workers to cope effectively with the psychological and emotional demands of work. Furthermore, high PSC implies that supervisors provide emotional support, understanding and validation of their workers which helps buffer against stressors at the workplace [5]. Besides, the presence of supportive supervisors or management contributes to workers’ mental well-being by reducing feelings of isolation, enhancing self-esteem, and promoting a sense of belongingness [7]. Moreover, in a high PSC context, supervisors are more likely to be responsive to workers’ needs and concerns, providing workers with essential resources and guidance that alleviate psychological distress [33, 34]. Thus, high PSC contexts encourage and empower workers by promoting assertiveness, resilience, and hope [39]. In such an environment, workers may feel more confident in expressing their needs, standing up for themselves, and seeking solutions to challenges. Consequently, this may lead to increased assertiveness, better coping mechanisms, and a more positive outlook on work-related issues [39].

The finding that the negative association between PSC and depressive symptoms is stronger for females than males highlight the potential of gender differences in the impact of PSC on mental health outcomes. This finding could be influenced by several variables including differences in socialisation, communication styles, and the importance of supportive relationships for women [36]. However, further research is needed to explore these gender-specific dynamics in more detail.

### The buffering effects of PSC

In a high PSC environment, job resources including job control and supportive leadership, are perceived as more beneficial and impactful [5]. Thus, PSC acts as an amplifier, enhancing the positive effects of these resources on workers’ mental well-being [43]. When workers perceive a supportive work environment, they are more likely to utilise job resources that are more effective in reducing psychological distress, increasing mindfulness, fostering personal hope, and mitigating the negative impact of job demands on depression and emotional exhaustion [7]. A high PSC context would create a sense of psychological safety, where workers feel comfortable expressing their concerns, reporting mistreatment, and seeking essential support [5]. This situation creates an environment where bullying, harassment, and other forms of workplace mistreatment are less tolerated, and thus, less occur [4]. The perception of high PSC buffers the adverse effect of workplace mistreatment on psychological well-being, post-traumatic disorder, stress, cognitive weariness and other psychological health problems to improve productivity and organisational image [4].

### PSC in specific workplaces

The workplace-specific findings underscore the intricate interplay between PSC and various professional domains, shedding light on the nuanced dynamics within diverse work settings [12, 35]. In the education sector, the observed reduction in psychological distress and emotional exhaustion among education workers in the presence of a positive PSC speaks to the profound impact of a supportive climate on educators’ well-being [4]. This suggests that cultivating an environment where educators feel psychologically safe translates into not only improved mental health but also potentially enhanced teaching effectiveness. Similarly, the buffering effect of PSC among police officers in Malaysia, mitigating the impact of job resources on psychological distress, implies that the nature of law enforcement work may be less psychologically taxing when embedded in a supportive organisational climate [7]. This finding holds implications for law enforcement agencies globally, urging a closer examination of the organisational factors influencing officers’ mental well-being.

In healthcare settings across different countries, the consistent positive outcomes, including decreased emotional exhaustion and stress, emphasise the universal importance of PSC in fostering a supportive environment for healthcare professionals [22, 30, 35]. The demanding and often emotionally charged nature of healthcare work makes the role of PSC in enhancing mental well-being particularly crucial. In the context of the general working population, the findings of reduced harassment, violence, and bullying in Australia underscore the broader societal impact of promoting a psychosocially safe work environment [10]. These results imply that organisational climates that prioritise employee well-being contribute not only to individual flourishing but also to creating healthier workplace cultures that extend beyond specific professions.

The enhanced mental well-being observed among construction workers in China suggests that the positive effects of PSC are not confined to traditional office settings [20]. In physically demanding and high-risk occupations, cultivating a supportive climate may play a pivotal role in mitigating the adverse psychological impacts of the job. Furthermore, the positive outcomes observed among attorneys in the USA and oil and gas workers in Malaysia highlight the relevance of PSC in diverse and high-pressure work environments [27, 28]. The findings imply that irrespective of the industry or professional demands, a psychosocially safe climate can act as a buffer against psychological distress.

### Practical implications for managers and organisations

Organisations and managers need to cultivate a supportive leadership style that emphasise open and bottom-up communication, approachability, and empathy towards workers [44]. Building positive relationships with workers and demonstrating genuine care is enhance PSC which contributes to creating a healthy and decent work environment where the psychological well-being of workers is prioritised. Furthermore, organisations should establish clear policies and procedures that explicitly address workplace mistreatment such as violence, bullying, harassment, discrimination, and abuse. These policies should be effectively communicated to all workers, reinforced and encouraged through training programmess. Organisations emphasising a zero-tolerance approach to workplace violence and harassment, have the potential of promoting a culture of respect and fairness, thereby promoting the health and well-being of their workers [45].

Managers and supervisors ought to undergo training on the significance of PSC and its relation to preventing mistreatment at the workplace. This training should concentrate on augmenting supportive leadership skills, promoting positive communication, conflict resolution, and creating an awareness of the impact of mistreatment on both individual and the organisation [46]. It is of utmost importance to institute confidential mechanisms for workers to report incidents of mistreatment without any apprehension of retaliation. Encouraging reporting can help identify and address mistreatment cases expediently. Managers should communicate the existence of reporting channels and ensure that workers feel secure and supported when reporting their concerns and seeking support and resources.

Managers possess a vital function in establishing a culture that highly regards respect, diversity, and inclusion. Through the cultivation of an inclusive work environment, wherein individuals are treated with dignity and fairness, managers can contribute to creating a high PSC context that minimizes the occurrences of mistreatment [46]. Managers should consistently evaluate and attend to work-related stressors that may lead to psychological distress, cognitive fatigue, emotional exhaustion, and depression. This can encompass the management of workload, provision of resources and support, and promotion of work-life balance. Organisations ought to allocate resources to workers’ well-being initiatives, such as mental health programmess, wellness activities, and workshops that develop resilience [46, 47]. Such initiatives may further reinforce psychological well-being, personal resilience, and hope among workers.

### Limitations and recommendations for future research

Most of the studies included this study were cross-sectional surveys which are usually affected by response bias, which may also affect the findings of this review. Using only papers published in the English language may affect the volume and depth of evidence retrieved for this review. There is limited evidence from continents such as Africa and South America that may skew the findings. However, authors used robust protocols to retrieve essential papers from 13 countries, screen papers, extract data and thematic analysis which may help in generalisation findings and make recommendations for future research and practice. Authors did not appraise the studies included in this scoping review. This poses a limitation as it may impact the overall quality and reliability of the included studies. Hence, caution should be taken when interpretating the findings and conclusion drawn from this review. Further research is needed to explore gender-specific dynamics in the influence of PSC on workplace mistreatment and mental health. A future systematic review is needed to estimate the practical effect of PSC on psychological well-being and workplace mistreatment.

## Conclusion

This review found that PSC has a negative association with workplace mistreatment such as bullying, harassment, violence, discrimination and abuse. Furthermore, the authors found that PSC has a positive association with psychological well-being, personal resilience and hope. PSC also has a negative association with psychological distress, stress, depression, cognitive weariness and emotional exhaustion, strongly establishing the buffering effect of PSC on worker health and well-being. The findings highlight the importance of fostering a supportive work environment and cultivating positive relationships between supervisors and employees. Workplaces or organisations should prioritise the training and development of supervisors to enhance their supportive skills, encourage respectful behaviour, and provide guidance on conflict resolution. By promoting a high PSC context, organizations can create a culture that discourages mistreatment, leading to increased employee well-being, job satisfaction, and productivity. Finally, organizations need to address factors that contribute to low PSC, such as ineffective leadership, lack of open bottom-up communication, or perceived unfairness. By identifying and addressing these issues, organisations can make practical steps towards creating a work environment that minimises mistreatment and promotes a positive workplace culture. Further research is needed to explore gender-specific dynamics in the influence of PSC on workplace mistreatment and mental health. A future systematic review is needed to estimate the practical effect of PSC on psychological well-being and workplace mistreatment in various and diverse organisational settings, especially in settings such as Africa and South America that have received limited research on PSC and its interplay with workplace mistreatment and mental health.

### Electronic supplementary material

Below is the link to the electronic supplementary material.

Supplementary Material 1

## Data Availability

All data generated or analysed during this study are included in this article and its supplementary file (Table S1).

## References

[1] ILO. Experiences of Violence and Harassment at Work: A first global survey. Experiences of Violence and Harassment at Work: A First Global Survey 2022.

[2] WHO, WHO/ILO ILO. accessed November 16,: Almost 2 million people die from work-related causes each year. WHO/ILO: Almost 2 Million People Die from Work-Related Causes Each Year 2021. https://www.who.int/news/item/16-09-2021-who-ilo-almost-2-million-people-die-from-work-related-causes-each-year (2021).

[3] WHO. ILO. WHO/ILO joint estimates of the work-related burden of disease and injury, 2000–2016: global monitoring report. Geneva: 2021.

[4] Dollard MF, Bakker AB (2010). Psychosocial safety climate as a precursor to conducive work environments, psychological health problems, and employee engagement. J Occup Organ Psychol.

[5] Dollard M, Dormann C, Idris M. Psychosocial Safety Climate. Vol. Eds. Springer International Publishing; 2019. 10.1007/978-3-030-20319-1.

[6] Hall GB, Dollard MF, Coward J (2010). Psychosocial Safety Climate: development of the PSC-12. Int J Stress Manag.

[7] Yulita Y, Idris MA, Dollard MF (2022). Effect of psychosocial safety climate on psychological distress via job resources, work engagement and workaholism: a multilevel longitudinal study. Int J Occup Saf Ergon.

[8] Pien LC, Cheng Y, Cheng WJ (2019). Psychosocial safety climate, workplace violence and self-rated health: a multi-level study among hospital nurses. J Nurs Manag.

[9] Arksey H, O’Malley L (2005). Scoping studies: towards a methodological framework. Int J Social Res Methodology: Theory Pract.

[10] Bailey TS, Dollard MF, McLinton SS, Richards PAM (2015). Psychosocial safety climate, psychosocial and physical factors in the aetiology of musculoskeletal disorder symptoms and workplace injury compensation claims. Work Stress.

[11] Law R, Dollard MF, Tuckey MR, Dormann C (2011). Psychosocial safety climate as a lead indicator of workplace bullying and harassment, job resources, psychological health and employee engagement. Accid Anal Prev.

[12] Bond SA, Tuckey MR, Dollard MF (2010). Psychosocial safety climate, workplace bullying, and symptoms of posttraumatic stress. Organ Dev J.

[13] Nguyen DTN, Teo STT, Grover SL, Nguyen NP (2017). Psychological safety climate and workplace bullying in Vietnam’s public sector. Public Manage Rev.

[14] Dollard MF, Dormann C, Tuckey MR, Escartín J (2017). Psychosocial safety climate (PSC) and enacted PSC for workplace bullying and psychological health problem reduction. Eur J Work Organizational Psychol.

[15] Klinefelter Z, Sinclair RR, Britt TW, Sawhney G, Black KJ, Munc A (2021). Psychosocial safety climate and stigma: reporting stress-related concerns at work. Stress Health.

[16] Escartín J, Dollard M, Zapf D, Kozlowski SWJ (2021). Multilevel emotional exhaustion: psychosocial safety climate and workplace bullying as higher level contextual and individual explanatory factors. Eur J Work Organizational Psychol.

[17] Vaktskjold Hamre K, Valvatne Einarsen S, Notelaers G (2023). Psychosocial safety climate as a moderator in role stressor- bullying relationships: a multilevel approach. Saf Sci.

[18] Afsharian A, Dollard M, Miller E, Puvimanasinghe T, Esterman A, De Anstiss H, et al. Refugees at work: the preventative role of psychosocial safety climate against workplace harassment, discrimination and psychological distress. Int J Environ Res Public Health. 2021;18. 10.3390/ijerph182010696. 10.3390/ijerph182010696 PMC8535317 34682442

[19] Idris MA, Dollard MF, Coward J, Dormann C (2012). Psychosocial safety climate: conceptual distinctiveness and effect on job demands and worker psychological health. Saf Sci.

[20] Xie L, Lin G, Hon C, Xia B, Skitmore M (2020). Comparing the psychosocial safety climate between megaprojects and non-megaprojects: evidence from China. Appl Sci (Switzerland).

[21] Zinsser KM, Zinsser A (2016). Two Case studies of Preschool Psychosocial Safety climates. Res Hum Dev.

[22] Afsharian A, Dollard M, Ziaian T (2019). Psychosocial Safety Climate. Psychosocial Saf Clim.

[23] Owen MS, Bailey TS, Dollard MF. Psychosocial Safety Climate as a Multilevel extension of ERI Theory: evidence from Australia. In: Siegrist J, Wahrendorf M, editors. Work Stress and Health in a globalized economy, aligning perspectives on Health, Safety and Well-Being. Springer International Publishing Switzerland; 2016. pp. 189–217. 10.1007/978-3-319-32937-6_9.

[24] Dollard MF, Opie T, Lenthall S, Wakerman J, Knight S, Dunn S (2012). Psychosocial safety climate as an antecedent of work characteristics and psychological strain: a multilevel model. Work Stress.

[25] Dollard, Karasek (2010). Building Psychosocial Safety Climate: evaluation of a socially coordinated PAR risk management stress Prevention Study. Contemp Occup Health Psychology: Global Perspect Res Pract.

[26] Mirza MZ, Memon MA, Dollard M. A time-lagged study on health-centric leadership styles and psychological health: the mediating role of psychosocial safety climate. Curr Psychol 2021:2021–3. 10.1007/s12144-021-02140-5.

[27] Gazica MW, Powers SR, Kessler SR (2021). Imperfectly perfect: examining psychosocial safety climate’s influence on the physical and psychological impact of perfectionism in the practice of law. Behav Sci Law.

[28] Mirza MZ, Isha ASN, Memon MA, Azeem S, Zahid M (2022). Psychosocial safety climate, safety compliance and safety participation: the mediating role of psychological distress. J Manage Organ.

[29] Inoue A, Eguchi H, Kachi Y, Tsutsumi A (2023). Perceived psychosocial safety climate, psychological distress, and work engagement in Japanese employees: a cross-sectional mediation analysis of job demands and job resources. J Occup Health.

[30] Afsharian A, Zadow A, Dollard MF. Psychosocial factors at work in the Asia Pacific. Psychosocial factors at work in the Asia Pacific: from theory to practice. Springer International Publishing Switzerland; 2016. pp. 187–201. 10.1007/978-3-319-44400-0_10.

[31] Idris MA, Dollard MF, Yulita (2014). Psychosocial safety climate, emotional demands, burnout, and depression: a longitudinal multilevel study in the Malaysian private sector. J Occup Health Psychol.

[32] Zadow AJ, Dollard MF, Mclinton SS, Lawrence P, Tuckey MR (2017). Psychosocial safety climate, emotional exhaustion, and work injuries in healthcare workplaces. Stress Health.

[33] Berthelsen H, Muhonen T, Bergström G, Westerlund H, Dollard MF (2020). Benchmarks for evidence-based risk assessment with the Swedish version of the 4-item psychosocial safety climate scale. Int J Environ Res Public Health.

[34] Havermans BM, Boot CRL, Houtman ILD, Brouwers EPM, Anema JR, Van Der Beek AJ (2017). The role of autonomy and social support in the relation between psychosocial safety climate and stress in health care workers. BMC Public Health.

[35] Mansour S, Tremblay DG (2019). How can we decrease burnout and safety workaround behaviors in health care organizations? The role of psychosocial safety climate. Personnel Rev.

[36] Zadow AJ, Dollard MF, Dormann C, Landsbergis P. Predicting new major depression symptoms from long working hours, psychosocial safety climate and work engagement: a population-based cohort study. BMJ Open. 2021;11. 10.1136/bmjopen-2020-044133. 10.1136/bmjopen-2020-044133 PMC8211051 34162636

[37] Bailey TS, Dollard MF, Richards PAM (2015). A national standard for psychosocial safety climate (PSC): PSC 41 as the benchmark for low risk of job strain and depressive symptoms. J Occup Health Psychol.

[38] Dormann C, Owen M, Dollard M, Guthier C (2018). Translating cross-lagged effects into incidence rates and risk ratios: the case of psychosocial safety climate and depression. Work Stress.

[39] Kwan SSM, Tuckey MR, Dollard MF (2016). The role of the psychosocial safety climate in coping with workplace bullying: a grounded theory and sequential tree analysis. Eur J Work Organizational Psychol.

[40] Siami S, Gorji M, Martin A (2022). Psychosocial safety climate and supportive leadership as vital enhancers of personal hope and resilience during the COVID-19 pandemic. Stress Health.

[41] Lawrie EJ, Tuckey MR, Dollard MF (2018). Job design for mindful work: the boosting effect of psychosocial safety climate. J Occup Health Psychol.

[42] Hall GB, Dollard MF, Winefield AH, Dormann C, Bakker AB (2013). Psychosocial safety climate buffers effects of job demands on depression and positive organizational behaviors. Anxiety Stress Coping.

[43] Loh MY, Idris MA, Dollard MF, Isahak M (2018). Psychosocial safety climate as a moderator of the moderators: contextualizing JDR models and emotional demands effects. J Occup Organ Psychol.

[44] Laloo E, Coman R, Hanley N, Bakand S (2023). The impact of leadership on the psychosocial safety climate of organizations: a scoping review. Int J Occup Saf Health.

[45] Zahlquist L, Hetland J, Skogstad A, Bakker AB, Einarsen SV (2019). Job demands as risk factors of exposure to bullying at work: the moderating role of Team-Level Conflict Management Climate. Front Psychol.

[46] Amoadu M, Ansah EW, Sarfo JO (2023). Influence of psychosocial safety climate on occupational health and safety: a scoping review. BMC Public Health.

[47] Amoadu M, Ansah EW, Sarfo JO (2024). Psychosocial work conditions and traffic safety among minibus and long-bus drivers. J Occup Health.

