# Supplementary material for: Preventing workplace mistreatment and improving workers’ mental health: a scoping review of the impact of psychosocial safety climate
Source: BMC Psychol. 2024 Apr 8;12:195. doi: 10.1186/s40359-024-01675-z (PMC11003102; doi:10.1186/s40359-024-01675-z)
Supplement: Supplementary file 1 — Supplementary Material 1 [file 40359_2024_1675_MOESM1_ESM.docx]

**Table S1: Extracted data from reviewed studies**

| **Authors/ country** | **Year of Publication** | **Purpose of study** | **Design** | **Population** | **Sample size** | **PSC measure** | **Outcomes** |
| --- | --- | --- | --- | --- | --- | --- | --- |
| [4]  Australia. | 2010 | To assess PSC as a precursor to conducive work environment, psychological health and engagement. | Longitudinal study | Education workers | 288 | PSC-4 | Psychological distress and emotional exhaustion (-s). |
| [7]  Malaysia | 2022 | To explore the effect of PSC on work investment and psychological health. | Longitudinal study | Polie officers | 392 | PSC-12 | PSC buffered the effect of job resources on psychological distress |
| [8]  China | 2019 | To assess the influence of PSC on workplace violence and self-rated health. | Cross-sectional survey | Healthcare workers | 1,690 | PSC-12 | Workplace violence (-s) |
| [10]  Australia | 2015 | To examine PSC as precursor to psychosocial work factors. | Longitudinal study | General working population. | 1,095 | PSC-12 | Harassment, violence, and bullying (-s). |
| [11]  Australia | 2011 | To assess PSC as a lead indicator of psychosocial work factors. | Cross-sectional survey | General working population | 1,134 | PSC-12 | Workplace harassment and bullying (-s).  PSC moderated the effect of bullying and workplace harassment on psychological health problem. |
| [12]  Australia | 2010 | The influence of workplace bullying and PTSD. | Longitudinal study | Police officers | 934 | 8-item PSC | Workplace bullying (-s)  PSC moderated the effect of workplace bullying on PTSD. |
| [13]  Vietnam | 2017 | To assess the role of PSC in a hostile workplace environment. | Cross-sectional survey | General working population | 274 | PSC-12 | Workplace bullying (-s). |
| [14]  Australia | 2017 | To assess the role of PSC in reducing workplace bullying and psychological health problem. | Longitudinal study | General population | 1,062 | PSC-12 | Workplace bullying (-s)  Psychological health (+s) |
| [15]  USA | 2021 | To examine the distinction between PSC and the Stigma in reporting stress. | Cross-sectional survey | General working population | 680 | PSC-12 | Bullying (-s). PSC played a moderating role in attenuating the effect of Stigma on bullying and burnout. |
| [16]  Spain | 2021 | To understand if PSC may explain emotional exhaustion. | cross-sectional survey | General working population. | 4,982 | PSC-4 | Bullying (-s)  Emotional exhaustion (-S). |
| [17]  Norway | 2023 | To examine the moderating role of PSC | Cross-sectional survey | General working population | 15,524 | PSC-8 | Workplace bullying (-s).  PSC moderated the association between role conflict and workplace bullying. PSC further moderated the association between role ambiguity and workplace bullying. |
| [18]  Australia | 2021 | To examine the role of PSC against workplace abuse and psychological distress. | Cross-sectional survey | Refuges workers | 117 | PSC-4 | Workplace abuse and discrimination (-s).  Psychological distress (-s) |
| [19]  Australia & Malaysia | 2012 | To examine the influence of PSC on job demands and psychological health. | Cross-sectional survey | Healthcare workers | 306 | PSC-12 | Psychological well-being (+s). |
| [20]  China | 2020 | To explore predictors of PSC in engineering construction projects | Cross-sectional survey | Construction workers | 624 | PSC-12 | Improved mental well-being (+s) |
| [21]  Australia | 2016 | To understand the PSC of pre-school environment. | Qualitative design | Pre-school teachers. | 16 | Interview items based on PSC-12 | Pre-school had policies and procedures that value teachers’ well-being and psychological safety. |
| [22]  Iran | 2019 | To examine the influence of PSC on mental health. | Cross-sectional survey | Healthcare workers | 247 | PSC-12 | Emotional exhaustion and psychological distress (-s). |
| [23]  Australia | 2016 | To examine PSC as an of effort-reward imbalance model extension. | Cross-sectional survey | General working population | 850 | PSC-12 | Psychological distress and depression (-s). |
| [25]  Australia | 2010 | To examine the role of PSC in managing stress. | Longitudinal study | School staff | 288 | 8-item PSC | Psychological distress and emotional exhaustion |
| [26]  Malaysia | 2021 | To examine the mediating role of PSC | Quasi-experiment | Oil and gas workers | 303 | PSC-12 | Psychological distress (-s). PSC mediates the association between health-centric leadership styles and psychological health. |
| [27]  USA | 2021 | To examine the influence of PSC on physical and psychological impact of perfectionism. | Longitudinal study | Attorneys | 176 | PSC-12 | Psychological distress (-s) |
| [28]  Malaysia | 2022 | To explore ethe impact of PSC on safety behaviours. | Cross-sectional survey | Oil and gas workers | 190 | PSC-12 | Psychological distress (-s) |
| [29]  Japan | 2023 | To examine PSC as predictors of psychological distress and work engagement | Cross-sectional survey | General Working Population | 2,200 | PSC-12 | Psychological distress (-s) |
| [30]  Iran & Australia | 2016 | To explore PSC in different cultural perspectives in Asia and Australia | Cross-sectional survey | Health workers | 496 | PSC-12 | Emotional exhaustion (-s). |
| [31]  Malaysia | 2014 | To explore the impact of PSC on job design and psychological outcomes. | Longitudinal study | Private sector workers | 370 | PSC-12 | Emotional exhaustion (-s) |
| [32]  Australia | 2017 | To examine the role of PSC in explaining emotional exhaustion and work injuries. | Longitudinal study | Healthcare workers | 214 | PSC-12 | Emotional exhaustion (-s). |
| [33]  Sweden | 2020 | To determine the benchmark for PSC for risk assessment. | Cross-sectional survey | General working population | 5,913 | PSC-4 | Stress (-s). |
| [34]  Netherlands | 2017 | To examine the association between PSC and stress | Cross-sectional survey | Healthcare workers | 277 | PSC-12 | Stress (-s) |
| [35]  Canada | 2019 | To examine the influence of PSC on fatigue and exhaustion. | Cross-sectional survey | Healthcare workers | 562 | PSC-12 | Cognitive weariness and emotional exhaustion mediate the relationship between PSC and safety workaround. |
| [36]  Australia | 2021 | To assess the association between PSC and degression. | Longitudinal study | Full time employees | 2,023 | PSC-12 | New major depressive symptoms (-s). The inverse relationship between PSC and depressive symptoms was stronger for females than males. |
| [37]  Australia | 2015 | To determine benchmark for PSC and the impact of PSC on reducing mental health issues at work. | Longitudinal study | General working population | 4,221 | PSC-12 | Job strain and depression (-s) |
| [38]  Australia | 2018 | To examine PSC as a risk factor for depression. | Longitudinal study | General working population | 1,905 | PSC-12 | Depression (-s) |
| [39]  Malaysia | 2016 | To examine the role of PSC in coping with workplace abuse. | Qualitative | General working population. | 20 | PSC-12 | Employees tended to voice in high PSC contexts, which led to swift resolution of  Bullying. In low PSC, bullying issues remained unresolved and led to turnover intentions and neglection. |
| [40]  Iran | 2022 | To assess the role of PSC as an enhancer of hope and resilience. | Cross-sectional survey | Healthcare workers | 623 | PSC-12 | PSC moderated the effect of supportive leadership on Personal hope. Personal resilience and hope (+s) |
| [41]  Australia | 2018 | To explore the impact of PSC on mindfulness. | Experiment | Education, healthcare and finance. | 57 | PSC-12 | As PSC was increased, job control was positively associated with everyday mindfulness at work. |
| [42]  Australia | 2013 | To test the moderating role of PSC on the effect of job demands and depression. | Cross-sectional survey | General working population | 2,343 | PSC-12 | PSC moderated the effect of job demands on depression. |
| [43]  Malaysia | 2018 | Testing PSC as a moderator of moderators. | Longitudinal study | Healthcare workers | 429 | PSC-12 | PSC was a strong moderator of emotional demand on psychological health problems |
| [44]  Ghana | 2020 | To explore the moderating role of PSC on the effect of psychosocial hazards and work engagement. | Cross-sectional survey | Bankers | 543 | PSC-12 | PSC moderated the effect of workplace bullying on work engagement. |
| (Dollard, Opie, et al., 2012)  Australia | 2012 | To assess PSC ad a determinant of work characteristics and psychological distress. | Longitudinal study | Healthcare workers | 365 | PSC-4 | Psychological distress (-s). |

-s, negative relation with PSC; +s positive relationship with PSC
